# The Role of Proteases in Determining Stomatal Development and Tuning Pore Aperture: A Review

**DOI:** 10.3390/plants9030340

**Published:** 2020-03-08

**Authors:** Dimitrios Fanourakis, Nikolaos Nikoloudakis, Polyxeni Pappi, Emmanouil Markakis, Georgios Doupis, Spyridoula N. Charova, Costas Delis, Georgios Tsaniklidis

**Affiliations:** 1 Department of Agriculture, School of Agricultural Sciences, Hellenic Mediterranean University, Estavromenos, Heraklion, 71500 Crete, Greece; dimitrios.fanourakis82@gmail.com; 2 Giannakakis SA, Export Fruits and Vegetables, Tympaki, 70200 Crete, Greece; 3 Department of Agricultural Sciences, Biotechnology and Food Science, Cyprus University of Technology, 3036 Limassol, Cyprus; n.nikoloudakis@cut.ac.cy; 4 Hellenic Agricultural Organization—‘Demeter’, Institute of Olive Tree, Subtropical Crops and Viticulture, Heraklion, 71307 Crete, Greece; polyxeni.pappi@nagref-her.gr (P.P.); markakis@nagref-her.gr (E.M.); gdoupis@gmail.com (G.D.); 5 Institute of Molecular Biology and Biotechnology, Foundation for Research and Development, Heraklion, 70013 Crete, Greece; charova@imbb.forth.gr; 6 Department of Biology, University of Crete, Heraklion, 70013 Crete, Greece; 7 Department of Agriculture, University of the Peloponnese, 24100 Kalamata, Greece; delis@us.uop.gr

**Keywords:** pore aperture, stomata, stomatal length, stomatal density, stomatal spacing, transpiration, water loss

## Abstract

Plant proteases, the proteolytic enzymes that catalyze protein breakdown and recycling, play an essential role in a variety of biological processes including stomatal development and distribution, as well as, systemic stress responses. In this review, we summarize what is known about the participation of proteases in both stomatal organogenesis and on the stomatal pore aperture tuning, with particular emphasis on their involvement in numerous signaling pathways triggered by abiotic and biotic stressors. There is a compelling body of evidence demonstrating that several proteases are directly or indirectly implicated in the process of stomatal development, affecting stomatal index, density, spacing, as well as, size. In addition, proteases are reported to be involved in a transient adjustment of stomatal aperture, thus orchestrating gas exchange. Consequently, the proteases-mediated regulation of stomatal movements considerably affects plants’ ability to cope not only with abiotic stressors, but also to perceive and respond to biotic stimuli. Even though the determining role of proteases on stomatal development and functioning is just beginning to unfold, our understanding of the underlying processes and cellular mechanisms still remains far from being completed.

## 1. Introduction 

In all living cells, the breakdown of functional proteins, as well as, the recycling of non-functional, misfolded or obsolete polypeptides to amino acids, are fundamental regulatory physiological and developmental processes, involving a diverse array of enzymes. These enzymes either selectively terminate proteins or generate biologically active peptides via cleavage. It is well known that this fundamental decomposition of proteins is carried out via either the ubiquitin/proteasome pathway or by inducing selective irreversible post-translational modifications, which in turn hamper protein functionality [1,2,3,4]. Proteolytic enzymes also participate in signaling pathways, which mediate diverse biological functions such as programmed cell death, as well as, plant responses to both biotic and abiotic stressors [4,5,6,7]. 

Plant genomes encode numerous proteases (also known as peptidases or proteolytic enzymes) that are structurally diverse enzymes despite having a common substrate-activity; namely they catalyze the hydrolytic cleavage of peptide bonds between peptide residues. After the initiation of the proteolytic mechanism, this process is mainly regulated by protease inhibitors [8]. Plant proteases are classified according to the MEROPS database (http://merops.sanger.ac.uk) in nine groups (the five major groups; serine, cysteine, aspartic, threonine, metalloproteases, as well as asparagine, glutamic, mixed and proteases with unknown catalytic type), based on the nature of the functional group of active sites that performs the (usually) selective hydrolysis of the peptide bonds [8,9,10]. 

**Figure 1:**
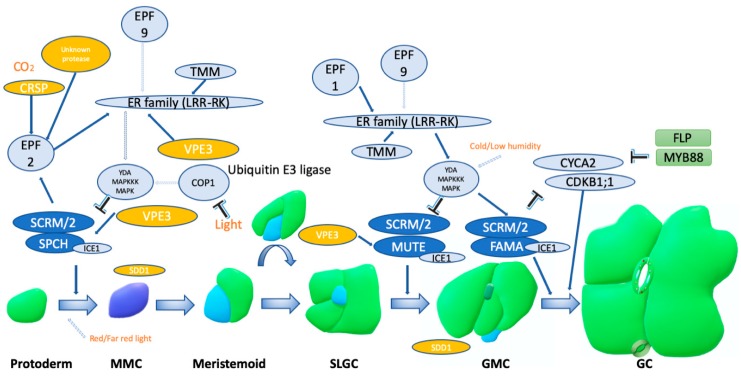
The main stomatal developmental pathway. The experimentally confirmed steps are shown as solid lines, and the steps that are yet unverified are shown as non-colored dotted lines. An arrow indicates a positive regulation, while a ‘T’ indicates negative regulation. Dark blue bubbles indicate critical controllers of the stomatal development, light blue bubbles indicate secondary regulators of the pathway, yellow bubbles mark the proteases that are involved in the pathway. Additional abbreviations: SCRM: Scream, YDA: YODA, MAPK: MAP Kinase, MAPKKK: MAPKK Kinase, SLGC: Stomatal lineage ground cell, FLP: Four Lips [16,17].

In higher plants, leaf gas fluxes primarily take place through stomata, which are actively regulated pores on the leaf surface [11,12,13]. The starting point of stomatal development is the protodermal stem cells which are differentiated in succession to Meristemoid Mother cells (MMC), Meristemoids, Guard Mother Cells (GMCs) and finally to Guard Cells (GC). This process is driven by three closely related transcription factors (SPEECHLESS (SPCH), MUTE and FAMA). The regulation of the SPCH, MUTE and FAMA mechanism is achieved with an array of other proteins and transcriptional factors exhibiting complex interactions allowing for its fine tuning according to the external stimuli. Indeed, hormonal and environmental signals are proven to affect this process by targeting specific of the plethora of regulators that affect the SPCH, MUTE and FAMA mechanism [14,15,16] (Figure 1). 

More specifically, a positive regulation towards the development of stomata has been confirmed for carbon dioxide, red light spectra, as well as the brassinosteroid signaling pathways. Furthermore, atmospheric air humidity and cold stress have also been implicated to control stomatal development and regulation [18].

Stomata play an essential role in the intake of CO_2_ for photosynthesis, and at the same time regulate transpirational water loss. A smaller portion of leaf gas fluxes occurs via the cuticle (passive regulation), a process that becomes increasingly important upon unfavorable (stress) conditions [19,20,21]. The stomatal cross-sectional area, where gas exchange occurs, is set by both stomatal opening (pore aperture) and stomatal anatomical features (size, density and patterning (spacing)) [22,23,24]. Consequently, leaf diffusive conductance involves both long-term processes and short-term dynamics [25,26]. Long-term processes refer to the establishment of stomatal anatomical features, as well as, the formation of the cuticle during leaf expansion (weeks); hence cannot be readily reset following this period [22,24,26]. On the other hand, short-term dynamics are resolved by tunings in pore aperture (seconds to hours), and thus are reversible [27,28,29]. This review examines the involvement of specific classes of proteases in the regulatory and signaling pathways that govern stomatal development, as well as, stomatal pore aperture tuning. 

## 2. Subtilisin-Like Serine Proteases

Subtilisin-like proteases (subtilases) are mostly endopeptidases, containing a group of the amino acids aspartate (Asp), histidine (His), and serine (Ser) in their active site, and are effectively folded by forming a β-sheet secondary structure comprising of seven beta strands. Some subtilases have shown high substrate specificity, participating in phytohormone precursors’ post-translational modification [30]. 

Three peptides have been shown to hold a predominant role in stomatal development (Epidermal Patterning Factor 1 (EPF1), Epidermal Patterning Factor 2 (EPF2), and Epidermal Patterning Factor Like 9 (EPFL9), also referred as STOM) [31,32]. EPF1 and EPF2 negatively regulate stomatal development by acting as ligands to activate the leucine-rich repeat receptor kinases (ER/LRR-RKs) [33], whereas, EPFL9 is a positive regulator, competing with EPF1 and EPF2 for LRR-RKs’ binding [33]. *EPF1*, *EPF2*, and *EPFL9* are initially translated as pro-peptides, the full activation of which is achieved via protease cleaving. The proteases activating the EPF1, EPF2, and EPFL9 via posttranslational modification have not yet been successfully identified. To date, the following two subtilisin-like proteases have been implicated in the stomatal development: the Stomatal Density and Distribution 1 (SDD1) and the CO_2_ Response Secreted Protease (CRSP or SBT5.2) [31].

The *SDD1* gene encodes a 775–amino acid protein in *Arabidopsis thaliana* (L.) Heynh., which exhibits homology with the S8 subtilisin-like serine protease [34]. *SDD1* gene expression is spatially and temporally limited to the stage of stomatal development in stomatal precursor cells. Transcripts were detected in meristemoids (stomatal initials) and GMCs, but were absent in mature stomata indicating a developmental rather than a constitutive role of SSD1 in stomatal formation [35]. In agreement to these results, Morales-Navarro et al. [36] reported increased transcription of the gene in growing tomato leaves suggesting the involvement of SDD1 in their development. SDD1 is a negative regulator of stomatal development, since it lessens both the formation of stomatal complexes and the number of stomata produced per stomatal complex [34]. Stomatal density was found to be higher (two to four-fold) in *sdd1–1* mutant as compared to the wild-type plants [33], whereas it was lower (two to three-fold) in the overexpressing lines [35]. A decrease in stomatal density, as a result of *SDD1* overexpression, has been generally correlated to enhanced water use efficiency and drought tolerance [37,38]. Moreover, SDD1 regulates the orientation of spacing divisions in neighboring cells [36]. In *sdd1–1* mutants, the principle of maintaining one epidermal cell spacing between adjacent stomata was violated, as a result of misoriented spacing divisions, thus forming stomatal clusters (i.e., two or more stomata touching) [34]. This contact between neighboring stomata has been related to pitfalls, including reduced carbon assimilation and impaired stomatal responses to external cues [22]. Moreover, similar functionality of SDD1 has been reported in *Arabidopsis* and tomato by Morales-Navarro et al. [36]. Although SDD1 strongly influences stomatal development, the proteolysis of EPF members by the SDD1 remains elusive [31,39].

The CRSP is another subtilisin-like protease involved in the control of stomatal development [40,41]. CRSP negatively regulates stomatal development under high CO_2_ concentration by processing the EPF2 precursor [39]. This processing results to blockage of the asymmetric divisions of the MMCs [42]. Wild-type *Arabidopsis thaliana* plants and the majority of plant species typically undergo a repression in stomatal development under elevated CO_2_ [41]. However, this response is disrupted in *crsp* mutants, which exhibit an inverted developmental response, namely producing more stomata at high CO_2_ levels [40]. EPF2 negatively regulates stomatal development [30]. Although CRSP has been shown to cleave EPF2, the effect of this cleavage on the interaction between EPF2 and LRR-RK remains unknown [40,41].

Senescence-Associated Subtilisin Protease (SASP) is another serine protease. It was recently demonstrated that apart from an elemental role in senescence, SASP is a key component in abscisic acid (ABA) signaling and drought tolerance. Indeed, SASP disintegrates Open Stomata 1 (OST1), an ABA signaling regulator, and in this way making stomata insensitive to ABA. Knocking down the *SASP* gene resulted to increased drought tolerance, since the stomatal response to stress-induced ABA was amplified [43,44]. 

## 3. Vacuolar Processing Enzymes (Cysteine Proteinases) 

According to MEROPS nomenclature, there are 63 subfamilies of cysteine proteinases that are subdivided into six groups (C, CA, CD, CE, CF and CH), making them one of the largest and most widely represented plant proteinase classes. Vacuolar processing enzymes (VPEs) are vacuolar localized cysteine proteinases that have been reported to hold multiple and important attributes in plant development, most notably regulating the mobilization of storage proteins in seeds. Moreover, VPEs possess critical roles in plant defense, as they are known to be involved in the process of programmed cell death under viral infection and hypersensitive responses. Recently, the VPEs’ role in the control of stomatal pore aperture during both pathogen attack and under abiotic stress (drought, salinity, low or high temperature) has been deciphered [45,46,47,48]. 

*Arabidopsis γ-vpe* mutant lines exhibited a drought tolerant phenotype. In this regard, it is interesting to note that *γ-vpe* knock-out mutants had a reduced stomatal opening, suggesting that this type of VPE is implicated in stomatal pore aperture regulation [49]. In rice, it was shown that the suppression of *OsVPE3* enhances salt tolerance by reducing vacuole rupture during programmed cell death, as well as by decreasing both leaf width and stomatal GC length [50]. Stomatal closure can be triggered by pathogens, pathogen-associated molecular patterns (PAMPs), and elicitors. VPEs are possibly involved in the control of the elicitor-induced stomatal closure by regulating NO accumulation in GCs [51], and thereby playing a key role in plant immunity.

Moreover, the suppression of *VPE3* led to reduced stomatal length in rice [50]. This effect was related to the downregulation of the expression levels of genes related to the stomatal development, namely: *Too Many Mouths (TMM)*, *Speechless (SPCH1)* and *Mute* [50]. Although this study clearly establishes a role of proteinases on determining stomatal length, the processes underlying this effect remain to be elucidated. 

Similarly, improved drought tolerance, owing to an enhanced control of water loss via stomata and increased ABA sensitivity, has also been reported following transgenic overexpression of *CYS4* (coding for a cysteine proteinase inhibitor) that targets both VPEs and Papain-like cysteine proteases in *Arabidopsis thaliana* and *Malus domestica* Borkh [52]. 

The direct inhibition of protease activity is expected to underlie these effects. Moreover, stomatal closure following elicitor inoculation was significantly inhibited by *VPE* silencing in *Nicotiana benthamiana* Domin [53]. The elicitor-triggered NO accumulation in GCs was also suppressed by VPE deficiency [46]. Taken together available data, suggests that VPE mediates the elicitor-induced stomatal closure by controlling the NO accumulation in GCs [46,51]. 

It appears that stomatal closure during the infection with various pathogens is a complicated cascade regulated by VPEs’ activity, which is influenced by NO signaling and can be triggered by PAMPs. Thus, it is suggested that VPEs possess a pivotal and multifunctional regulatory role in the initial defensive physiological reactions against pathogens [6,48,53]. 

## 4. Papain-Like Cysteine Proteases

Papain-like cysteine proteases (PLCPs), featuring a nucleophilic cysteine thiol at the active site, are coded by a multigene family (with at least 31 members in *Arabidopsis*). These genes contribute to a plethora of physiological procedures, such as seed germination, anther development, programmed cell death, senescence, abiotic stress responses and plant innate immunity system [54]. In the apoplast, the vast majority of proteases, recognizing different pathogen types and transmitting the respective messages, belongs to PLCPs. However, different PLCPs enable separate signaling pathways initiating the appropriate innate immunity response [6]. 

Further evidence on the role of proteases in stomatal regulation and development comes from recent work in barley [55]. Knock-down lines with reduced transcription of two Papain-like cysteine protease genes (*PAP-1* and *PAP-19*), exhibited differential stomatal development and functionality as compared to wild-type plants. Even though both *pap-1* and *pap-19* knock-down lines exhibited a decrease of stomatal pore area, still, only the stomatal pore of *pap-19* plants was significantly larger than the wild-type. According to the same report, *pap-1* and *pap-19* lines differentially responded to biotic stimuli. Moreover, the phytohormonic equilibrium (especially jasmonic acid levels) under drought stress was different between knock-down lines and wild-type plants, thus suggesting that a delicate crosstalk among phytohormones, proteases and stomatal regulation occurs under stress. 

A large number of pathogens uses stomatal pores as an entrance in order to colonize inner leaf tissues. For instance, several pathogens such as *Plasmopara viticola* and *Puccinia* Pers. fungal species [56,57,58] specifically internalize leaves only through stomata. In addition, stomatal pores can also serve as the occasional entrance for many other pathogens, such as the bacteria *Xanthomonas campestris* pv armoraciae and *Pseudomonas syringae* [59]. While ABA appears to be a key regulator in stomatal closure, both salicylic acid (SA) and hydrogen peroxide, which are produced under several modes following pathogen attack and stress stimuli, are also involved. Indeed, SA and hydrogen peroxide have been both shown to halt pathogen penetration inside the plant body [59,60,61]. Recently, Ziemann et al. [62] reported that through the activity of a Papain-like cysteine protease, the immune signaling peptide 1 (ZIP1) is matured from its pro-peptide, and serves as an activator of SA signaling via transcriptional upregulation. 

## 5. Aspartic Proteases

In *Arabidopsis thaliana*, an Aspartic protease (Aspartic Protease in Guard Cell 1; ASPG1) with a preferred localization in the stomatal GCs, has been implicated in ABA sensitivity acting competitively to the Small ubiquitin-like modifier (SUMO) proteases. The overexpression of ASPG1 gene resulted in faster stomatal closure under drought stress by enhancing the ABA sensitivity of GCs [63]. Correspondingly, another aspartic protease (APA1) has also been implicated in drought tolerance by exhibiting a similar activity to ASPG1. The overexpression of *APA1* gene resulted to increased drought tolerance, as plants exhibited reduced stomatal index and thus reduced water loss [64]. Moreover, Aspartic protease (AP17) has also been demonstrated to positively affect both ABA and antioxidant responses under stress, while it negatively affected stomatal pore aperture in grape vine [65]. In addition, the aspartic protease Constitutive Disease Resistance 1 (CDR1) has a fundamental role in the initiation of SA-related signaling under pathogen attack that stimulates the innate immunity reactions [66]. While stomatal closure is one of the most characteristic innate immunity reactions, still the association between CDR1 activity and stomatal functionality is at the side of expectations, though this hypothesis remains to be experimentally addressed. 

## 6. Ubiquitin-Mediated Proteasomal Protein Degradation and Ubiquitin-Like Modifiers

Ubiquitin-mediated proteasomal protein degradation is an important mechanism to control protein load in the cells. Ubiquitin, a regulatory protein that consists of 76 amino acids, binds to lysine residues of proteins, and usually promotes its degradation through the 26S proteasome complex (a process known as “ubiquitination”). Abnormal, misfolded proteins, as well as, regulators of many processes are marked, and then degraded by the ubiquitin-proteasome system. This process allows cells to regulate the response to cellular level signals and altered environmental conditions [67]. The ubiquitin-mediated proteasomal degradation system has a key role in abiotic stress feedback, immunity, and hormonal signaling by interfering with key components of these pathways.

*SPCH, MUTE*, and *FAMA* transcription factors act in conjunction with several other proteins and transcription factors, orchestrating stomatal formation and differentiation [68]. This major regulatory system interacts with other transcription factors, such as the *Inducer of CBF Expression (ICE)*, during plant development with defined roles under specific environmental conditions [69]. Proteases’ function also interplays with light spectra. Under dark, in the abaxial (lower) *Arabidopsis* epidermal cells, ICE is degraded by an E3 ubiquitin ligase (Constitutive Photomorphogenic 1-COP1), influencing stomatal development. Also, it was demonstrated that the activity of COP1 was suppressed by blue, red and far-red light. Moreover, COP1 interacts with phytochrome A, phytochrome B and cryptochrome 1, indicating that this system is fundamental for plant development in the ever-changing light environments [67,68]. Moreover, the involvement of another protease (E3 ubiquitin ligase-HOS1) in photoperiodic flowering and its similar mechanism of action in conjunction with phytochrome B, suggests that it can also be involved in stomatal development alongside to COP1 [70].

Additionally, the *ethylene response transcription factor* (*ERF*) family mediates a large number of plant developmental or stress-induced responses. It is documented that the NO sensing-induced stomatal closure, under specific environmental stimuli, is promoted by the degradation of a group of ERF factors. This degradation is achieved through the 26S proteasome and via the activity of a specialized E3 ubiquitin ligase [71,72].

The SUMO (small ubiquitin-like modifier) mechanism includes both specific SUMO ligases and SUMO proteases. Particularly, the SUMO procedure regulates the functionality of specific target proteins by attaching or cleaving ubiquitin-like polypeptides, and in this way regulates their activity, localization or integrity. The SUMO-tagging, however, is not employed for the protein proteasome disintegration, but only for the modification of the protein activity. Sumoylation appears to be a cornerstone regulatory mechanism for plant development and for the response to environmental stimuli [73,74].

In *Arabidopsis*, the silencing of two genes, coding for SUMO proteases (OTS1 and OTS2)*,* resulted in increased stomatal pore aperture under decreased water potential, while notably leaf transpiration performance remained relatively unaffected. Moreover, *ots1* and *ots2* knocked-down lines that germinated in a medium containing 1 μM ABA resulted in plants having larger stomatal pore size than wild-types [73]. In rice on the contrary, transgenic seedlings with altered levels of transcription (knocked-down or over-expressing) OTS1 SUMO protease did not exhibit differences in stomatal density. Still effects on the stomatal functionality were detected, since OTS1-overexpressing plants lost more water after a drought incident in comparison to wild-type and *ots1*-RNAi lines [75].

Recently, Orosa et al. [76] reported that the activity of a specific PAMP bacterial related sensor, Flagellin Sensing 2 (FLS2), is regulated by a class of SUMO proteases that target the receptor. FLS2 removes small ubiquitin-like SUMO chains, thus, adjusting the signal strength and consequently the immune response. Apart from the other immune responses triggered by the receptor, FLS2 also mediates the PAMP-associated stomatal closure. This is achieved even under high light levels that favor stomatal opening by inhibiting the influx K^+^ channels in the stomatal GCs with interrelation of ABA signaling [77,78]. Finally, it should be noted that several E3 ubiquitin ligases and F-Box proteins greatly influence stomatal functionality, especially under stress. However, since the activity of these enzymes differs from proteolysis [79,80], it falls out of the scope of this study.

## 7. Conclusions and Perspectives

Proteases selectively cleave proteins. Their involvement in controlling stomatal development and adjusting pore aperture is critically surveyed. Direct or indirect effects of proteases on stomatal index, density, spacing and size have been observed suggesting that proteolysis is a critical regulator of stomatal development. Moreover, several proteases have also been implicated to orchestrate the stomatal response to various biotic and abiotic stimuli. As stomata can be a critical entrance point for pathogens, while the precise regulation of stomatal functionality is fundamental for plant physiological adaptations under abiotic stress conditions, the alteration of stomatal activity exerts a large impact on plant ability to cope not only with abiotic, but also with biotic stressors. The determining role of proteases on several aspects of stomatal development and functioning has been established, though the underlying processes are still not fully deciphered. For example, the link between stomatal defense and the activity of metalloproteinases, especially of the apoplastic Matrix Metalloproteinases, which are known to participate in signaling and defense responses under pathogen attack, is to our knowledge yet undiscovered [81,82]. The emergence and use of powerful mutagenesis techniques, such as Clustered Regularly Interspaced Short Palindromic Repeats/CRISPR-associated endonuclease 9 (crispr/cas9), can generate an array of mutants in order to elucidate the mechanisms and the molecules participating into stomatal organogenesis and function. Moreover, transcriptional regulation of SPCH, MUTE and FAMA under diverse biotic or abiotic stresses could identify possible crosslinks of unidentified proteases regulating the development of stomata, and thus approaching a more complete picture of the underlying processes.

## Abbreviations

ABA, Absisic Acid; Asp, aspartate; ASPG1, ASPARTIC PROTEASE IN GUARD CELL 1; COP1, CONSTITUTIVE PHOTOMORPHOGENIC1; CDR1, Constitutive Disease Resistance 1; CRSP, CO_2_ RESPONSE SECRETED PROTEASE (also referred as SBT5.2); EPF, EPIDERMAL PATTERNING FACTOR; EPF1, EPIDERMAL PATTERNING FACTOR 1; EPF2, EPIDERMAL PATTERNING FACTOR 2; EPFL9, EPIDERMAL PATTERNING FACTOR LIKE 9; ERF, ethylene response transcription factor; FLS2, Flagellin Sensing 2; GC, Guard Cells; GMC, Guard Mother Cells; His, histidine; ICE, Inducer of CBF Expression; LRR-RK, leucine-rich repeat receptor kinase; MMC, Meristemoid Mother cells; OsSPCH1, SPEECHLESS; OST1, Open Stomata 1; OsTMM, TOO MANY MOUTHS; PAMPs, pathogen-associated molecular patterns; PLCPs, Papain-like cysteine proteases; SA, Salicylic Acid; SASP, Senescence-Associated Subtilisin Protease; SDD1, STOMATAL DENSITY AND DISTRIBUTION 1; Ser, serine; SUMO, Small ubiquitin-like modifier; SPCH, SPEECHLESS; VPE, vacuolar processing enzyme.
